# The eye of the beholder: how do public health researchers interpret regression coefficients? A qualitative study

**DOI:** 10.1186/s12889-023-17541-3

**Published:** 2024-01-02

**Authors:** Taya A. Collyer

**Affiliations:** 1 Monash University - Peninsula Clinical School, Central Clinical School, 2 Hastings Rd, 3199 Frankston, VIC Australia; 2 National Centre for Healthy Ageing, Melbourne, VIC Australia

**Keywords:** Biostatistics, Statistical knowledge, Statistical practice, Regression, Health equity, Sociology of science

## Abstract

**Background:**

Calls for improved statistical literacy and transparency in population health research are widespread, but empirical accounts describing how researchers understand statistical methods are lacking. To address this gap, this study aimed to explore variation in researchers’ interpretations and understanding of regression coefficients, and the extent to which these statistics are viewed as straightforward statements about health.

**Methods:**

Thematic analysis of qualitative data from 45 one-to-one interviews with academics from eight countries, representing 12 disciplines. Three concepts from the sociology of scientific knowledge and science studies aided analysis: Duhem’s Paradox, the Agonistic Field, and Mechanical Objectivity.

**Results:**

Some interviewees viewed regression as a process of discovering ‘real’ relationships, while others indicated that regression models are not direct representations, and others blended these perspectives. Regression coefficients were generally not viewed as being mechanically objective, instead interpretation was described as iterative, nuanced, and sometimes depending on prior understandings. Researchers reported considering numerous factors when interpreting and evaluating regression results, including: knowledge from outside the model, whether results are expected or unexpected, ‘common-sense’, technical limitations, study design, the influence of the researcher, the research question, data quality and data availability. Interviewees repeatedly highlighted the role of the analyst, reinforcing that it is researchers who answer questions and assign meaning, not models.

**Conclusions:**

Regression coefficients were generally not viewed as complete or authoritative statements about health. This contrasts with teaching materials wherein statistical results are presented as straightforward representations, subject to rule-based interpretations. In practice, it appears that regression coefficients are not understood as mechanically objective. Attempts to influence conduct and presentation of regression models in the population health sciences should be attuned to the myriad factors which inform their interpretation.

## Background

Statistical methods comprise the heart of much population health research [1]. However, statisticians and methodologists are involved ‘sporadically’ [2], and calls for urgent improvement in statistical practice have persisted for decades [3, 4].

To date, efforts to improve statistical practice in public health research have not included direct investigation of the views and beliefs which underpin established practice [5]. Many studies evaluate statistical *knowledge* among particular groups [6, 7] or describe reporting practices in particular journals [8, 9]. Several highly-cited studies describe widespread error or misunderstanding in population health research [10, 11] including among clinicians [12], and statistics instructors [13–15]. Studies quizzing participants on the correct interpretation of a particular statistic (a method pioneered by Oakes [16]) routinely report error rates greater than 80% [15, 17]. Whilst documenting *that* misunderstanding and error are widespread is important, this alone will not occasion cultural change. Also needed are studies which aim to uncover *why* researchers do what they do with statistical methods. Additionally, framing biostatistical practice as simply correct or incorrect leaves little room for exploring diverse ideas held by researchers, including diverse understandings regarding what correctly-implemented analyses signify for the health of populations.

Current empirical approaches to statistical practice in the population health sciences have typically involved description of observed practice by statistical experts. To borrow from social anthropologist Clifford Geertz, such description is quite likely to be ‘systematically deaf’ to the realities researchers face in their day-to-day work [18]. Quoting Geertz further, rather than describing practice from a distance, it may be analytically fruitful to move beyond description, to interrogate *what researchers they think they are doing* via statistical analyses.

In this paper I present an example of this approach, focused on the ways researchers interpret and understand regression coefficients; nearly ubiquitous in quantitative health-related research [19]. The use of regression techniques imposes a number of statistical assumptions, which are not routinely discussed but can powerfully shape conclusions drawn from data [20]. To explore variation in the way regression coefficients are understood to represent facts about health, I employ three concepts from the Sociology of Scientific Knowledge and Science and Technology Studies; Duhem’s Paradox [21], the Agonistic Field [22], and Mechanical Objectivity [23]. I introduce these concepts, below.

### Duhem’s paradox & the agonistic field

Scientific experiments might be understood as self-contained endeavours, simple tests of a motivating hypothesis via new data. However, when faced with unexpected results, researchers cannot definitively judge whether these constitute novel findings, or suggest an experimental design flaw. This is Duhem’s ‘paradox’ [21], the way in which results from scientific experiments cannot be fully interpreted without reference to established propositions, theory, and other external information.

Interpretation of beta coefficients (statistics representing how some outcome tends to change over different levels of an explanatory variable) may represent an arena within which Duhem’s paradox is encountered in population health research. Unexpected effect sizes, or effects in unexpected directions might suggest new discoveries, or may indicate programming errors, confounding, or bias. The possibility that coefficients are distorted by confounding and/or bias is widely discussed in epidemiology textbooks [24]. Multivariable regression is frequently presented as a solution, but criteria for deciding when to initiate or cease investigation into such concerns rarely moves beyond advice to consider ‘all known and unknown confounders’ [24–26]. Formal frameworks have been presented to guide researchers in adjudication of research results, however these mostly relate to reasoning surrounding causal interpretation [27]. Not all population health research is causal, and formal causal frameworks are not universally employed. Therefore, while the need to progress to multivariable analysis is widely accepted, the specific ways researchers navigate uncertainty within multivariable analyses remain unclear.

In many studies the answer to a research question takes the form of a single regression coefficient. It is on the basis of regression coefficients that researchers argue, for example, that a treatment works, or that a particular population is at increased risk of disease. However, the status of these statistics as wholly constituting ‘answers’ is unclear. Theorists Latour and Woolgar [22] suggest the on-screen appearance of coefficients is unlikely to represent the end of analysis and interpretation, it is more likely the beginning of what they term an ‘agonistic process’, the shepherding of a new statement through a gauntlet of suspicion and scepticism. In the moment when results first appear,

members of the laboratory are unable to tell whether statements are true or false, objective or subjective, highly likely or quite probable. While the agonistic process is raging, modalities are constantly added, dropped, inverted and modified (p. 150).

In Latour’s observations of a Nobel-prize-winning lab, discussions about what might undermine or reinforce a particular experiment’s integrity were frequently disorderly, and circular. The journey from speculation to fact was characterized by addition and subtraction of *modalities* (qualifiers tying results to local circumstances) limiting their generalizability. In epidemiology, suspicion about confounding or selection bias may represent such modalities. For example, in early analysis a coefficient might ‘suggest’ a relationship (or ‘trend’) between exposure and outcome, or be limited to a particular population, at a particular time. Latour and Woolgar argue that whilst such concerns persist, the agonistic process continues and claims cannot stabilize as facts.

Facts (modality-free statements which survive agonistic processes) are very rare compared with the large volume of speculative claims which emerge and perish during analysis, and are special by virtue of their being understood as true by ‘all concerned parties’ (members of the relevant specialty). In population health, the statement “smoking causes lung cancer” is one such example: this statement is modality-free, it is understood to apply in all places, at all times, by all concerned parties.

Duhem’s paradox and the agonistic field help to conceptualise the role of regression coefficients in knowledge construction within population health; coefficients may represent numeric proto-statements about health, emerging into an agonistic field, subject to modalities and the inherent uncertainty implied by Duhem’s paradox.

### Mechanical objectivity

Within life-sciences, knowledge claims arising from mechanistic processes tend to be valued above claims arising from judgement or subjective opinion. Daston & Galison [23] detail the origin of this development in the 17th Century, as mechanical, automated images replaced artistic renderings in scientific publications. Mechanical instruments entered laboratories, and were “patient, indefatigable, ever alert, probing beyond the limits of the human senses”. By the 19th Century, *mechanical objectivity* emerged as a dominant scientific ideal, and automated imaging technologies such as x-ray eliminated human intervention from scientific representation. Selectivity and judgement were reframed as “temptations requiring mechanical or procedural safeguards” [23].

Automated statistical technologies emerged during the 20th century, and the impact of mechanical and procedural safeguards in biostatistics is one of the biggest issues facing the field. In particular, Null Hypothesis Significance Testing (NHST), a mechanistic procedure which eliminates expert judgement from inference [28], now dominates quantitative analyses of health-related data. Impassioned commentary and debate surrounds NHST’s continued use [4], with attempts to remove and replace it generally regarded as unsuccessful [29, 30]. Regression coefficients are very often the focus of hypothesis tests, and it may be that mechanized, automated statistical procedures possess the superhuman epistemic aura of mechanized images documented by Daston and Galison [23].

Crucially, however, automated scientific representations are rarely mechanically objective in practice. The earliest radiographers noted that X-ray equipment was sensitive in numerous ways, and had to be setup “just-so” [23]. Thus, while mechanically-generated representations may be *perceived* as being judgement-free, in practice they require substantial expertise to generate and interpret, they require a ‘seeing eye’ [23].

The privileging of quantification in health-related research has attracted multiple, sustained lines of criticism, notably drawing upon feminist [31] and post-colonial [32] frameworks. However, it has not been empirically established that researchers in the population health sciences view statistical results as straight-forward, objective renderings of nature. Daston and Galison’s work suggests that despite being mechanical in their generation, these outputs may be viewed as sensitive and deceptive renderings of complex health phenomena, requiring skilled interpretation.

Drawing on the concepts just outlined, in this paper I ask whether researchers appear to be navigating Duhem’s paradox in their interpretation of regression coefficients, and whether such results are reported as being shepherded through something like an agonistic field. I also ask to what extent mechanically-generated statistical output is received as the straightforward ‘word of nature’ [23] or is interpreted through the critical lens of subject-matter expertise by population health researchers.

## Methods

### Data collection overview & interviewee characteristics

One-to-one qualitative interviews were conducted with 45 population health researchers. Interviewees were sampled from 250 potential researchers, identified via bibliometric analysis of the global health inequalities and disparities research field (described elsewhere [33]). Health equity research is a useful sampling frame because it is comprised of researchers from diverse disciplines tackling similar research questions. Briefly, the prior bibliometric analysis drew upon 29,212 publications to produce an author-level map of the research area, which was then divided algorithmically into 8 sub-networks of researchers who tend to cite one another. On inspection, these clusters were found to represent geographic, institutional, and disciplinary research communities. Sampling for interviews was purposive, jointly informed by five recruitment priorities: (i) representing all regions of the bibliometric network, (ii) representing a range of disciplines, (iii) representing diverse geographic locations, (iv) representing a range of career stages, and (v) representing a mixture of very high-profile scholars and those with lower public profiles. In total, 113 researchers were contacted in 2018–2019, and 45 ultimately participated (response rate 40%). Seven of the eight citation clusters are represented, with at least three individuals interviewed from each cluster. Despite repeatedly contacting all researchers within the ‘Cancer Disparities, Administrative Reporting’ cluster, this group is not represented. Two-thirds of eligible researchers were located in the US and UK [33], and 60% of interviewees are likewise located in these two countries. Almost all countries are included, with the exception of South American researchers (located in Brazil and Chile) who comprised 2% of the bibliometric network, and researchers located in Asia (Japan and South Korea) who also comprised 2%. Interviews were conducted via in-person meetings (n = 15), phone-calls (n = 3) and video-calls (n = 28) and no incentive was provided to participants. Interviewees represent twelve disciplines and eight countries (see Table 1, and further detail on interviewees’ first and last degrees previously published [33]). 20/45 interviewees (44%) identified as female and all interviewees hold a PhD. Interviewees are established academics, with 40/45 holding associate professorial appointments or higher. To preserve anonymity of interviewees, they are referred to by their appointment and country. Where relevant to analysis, interviewees’ disciplinary trainings are included alongside select, brief extracts.

To support analysis, I separated interviewees into four groups reported in Table 1. These groups reflect the extent to which the interviewee engages in hands-on analysis, on the basis of data arising at all parts of the interview. I did not have sufficient data to classify one interviewee.

**Table 1:** Interview location and discipline at time of interview conduct

	N
**Country**	
UK	14
USA	13
Netherlands	5
Australia & New Zealand	5
Canada	3
Spain	3
Scandinavia	2
Total	45
**PhD Discipline**	
Epidemiology	9
Economics	5
Public Health	4
Biostatistics	3
Geography	3
Medicine	3
Sociology	3
Anthropology	2
Demography	2
Nursing	2
Psychology	2
Social Science^a^	7
Total	45
**Use of statistical methods**	
Mostly does own quantitative analysis	18
Mostly delegates analysis to others	20
Statisticians	3
Qualitative researchers who do not conduct or delegate quantitative analyses	3
Unknown	1

^a^To prevent interviewees being identifiable, social sciences (other than sociology, anthropology, economics and demography) are reported together

Interviews were semi-structured, following a thematic schedule lasting 30–90 min. Interviews formed part of a broader project aiming to illuminate disciplinary differences among population health researchers. Five themes were covered, including: Interviewees’ own disciplinary training and identity, views regarding the key determinants of health, experience collaborating with researchers from other disciplines, perceptions of excellent and weak empirical work, and specific discussion of regression methods.

### Interview schedule development and data analysis

Support in the literature to guide questioning on the regression methods theme was scarce. Interview content was therefore developed by drawing upon my own professional experience as a biostatistician, rather than previous empirical work. This theme tended to include three general lines of questioning: (i) ‘If a colleague asked, “what do regression models do?” how would you respond?’ (ii) ‘Do regression models represent reality?’ And (iii) An invitation for interviewees to describe whether they view regression models as representing discovery, or construction. A pilot phase of four interviews helped to refine these lines of questioning. It was immediately obvious that interviewees were reluctant to discuss statistical issues ‘with a statistician’, and so I added reassurance that I was not testing or quizzing interviewees, and adapted the final line of questioning (described more fully in Results). Interviews were recorded and transcribed verbatim by the author. Transcripts were anonymized in consultation with interviewees (19/45 requested the opportunity to review transcripts for accuracy and anonymity), before being coded for thematic analysis in R (Version 4.0.0 [34], Rstudio Version 1.2.5 [35]) using the RQDA package Version 0.3.1 [36]. In accordance with the consent form and ethics approval, transcripts were seen only by the author and interviewee. The project received ethical approval from the University of Edinburgh, School of Social and Political Science.

Following initial thematic analysis, I reviewed theories and concepts from the Sociology of Scientific Knowledge, Science and Technology Studies, History and Philosophy of Science literatures which shed light on both how and why scientists’ interpretation of mechanically generated statistical output might be expected to vary. The three selected concepts were then integrated into the final analysis, allowing me to interpret interview data through the lens of these concepts.

Highlighting epistemological diversity is viewed as controversial, even dangerous in some scientific settings [37, 38]. In describing diverse approaches I do not imply that all approaches are correct, or that no approach is correct [39]. Researchers who agree on technical definitions may disagree regarding what has been learned about health once a coefficient is estimated. If present, such diversity has implications for efforts to alter statistical norms, and this is the motivation for the following analysis.

## Results

### Regression as knowledge discovery and/or construction

Pilot interviewees indicated that direct questions regarding the epistemic status of coefficients were difficult to answer. I therefore invited the 28 interviewees who had conducted regression analyses to position themselves along a spectrum: Viewing regression modelling as knowledge discovery, knowledge construction, or something in between. Interviewees might consider the coefficient as something which is *discovered* by eliminating bias and revealing ‘the truth’, or as something actively *constructed*, subject to myriad caveats. In responding, nine interviewees articulated a clear position, three opted not to answer and sixteen discussed how they generally view regression coefficients corresponding with reality.

Several interviewees struggled to answer. Four interviewees from diverse disciplines (located in Oceania, the USA, Europe and UK) began by noting they had not considered the issue before, their responses are detailed in Table 2.

**Table 2:** Considering the epistemic status of regression coefficients was novel for some interviewees

Question: “Is fitting a regression model a process of discovering a set of relationships which really exist, or a process of constructing a representation of what might be real?”	
Interesting! That is interesting. [Long pause]. I don’t know. I don’t know. […] I definitely understand both sides but I’m not sure I’ve ever thought about it in that way.	Health Promotion PhD
Hmmm. I’m not sure I have a preconceived response to that question.	Sociology PhD,
[Long pause]. I haven’t been asked that question before.	Economics PhD
[Pause]. I haven’t thought about that. […] It’s something to think about.	Epidemiology PhD

That senior researchers from such varied backgrounds and locations report not having deeply considered the epistemic status of a statistic central to research about health is itself interesting. Although only four interviewees were open about never having considered the question, it was clear the majority of interviewees did not have a prepared response.

### Regression as discovery

Interviewees leaning toward the idea that models facilitate discovery tended to reminisce about particular, surprising results:

With our stuff on [blank] inequalities, the model was the discovery. I wasn’t expecting that [result], […] it was a bit like an archeological process, sweeping away the soil and suddenly, there’s excalibur (Professor, UK).

For this researcher, a coefficient suggested a revised interpretation of a commonly-understood relationship, and this interpretation was accepted as corresponding with reality rather than as reflecting a flaw in the study. But, interpretation of unexpected results was not always so clear:

I am always a bit cautious. If you get something that looks weird, check it and see, maybe test it using a split sample, or another dataset, or something. It should make sense… But I’m not ruling out the possibility that you find something that is completely left field. […] They [unexpected results] may or may not be [correct], but at this stage you don’t know what they are (Professor, UK).

This interviewee articulated Duhem’s paradox quite neatly, emphasizing inability to know what unexpected results mean at the time they arise. Faced with uncertainty, knowledge from outside the model (another dataset) is necessary to inform interpretation. For this interviewee, despite mechanistic generation, regression coefficients do not speak the ‘words of nature’ [23] but are somewhat garbled statements, to be treated with caution and cross-checked against other sources. Consistent across interviews was the sense that such confirmatory investigations do not *provide* an interpretation, they inform the researchers’ interpretation. The researcher decides whether a result has meaning (represents ‘excalibur’, new knowledge) or is an artefact, to be dismissed or re-estimated. This contrasts with the way regression coefficients are presented in teaching materials, as fairly straightforward estimates of underlying relationships.

Interestingly, not all coefficients are treated with equal scepticism, and interpretation seemed to depend on researchers’ expectations and prior knowledge. Comments like the above that coefficients “should make sense” were widespread, seeming to suggest that results fitting with researchers’ expectations are somehow different to results which don’t:

My radar would be up more if it [the estimated effect] is an unexpected direction, or it was significant when I thought it wasn’t going to be. You know, so I guess once that happened, I don’t just take it at face value that “Yep, that’s it”. But I don’t tend to do that for results that are, sort of, more expected (Professor, USA).

If interpretation varies depending on whether results align with researchers’ prior understanding, regression coefficients are not mechanically objective, but require expert judgement and a ‘seeing eye’ [23]. The interviewee above went on to say:

I think you have to take those [unexpected] results, but view them from a lens of common-sense.

As ‘common-sense’ is located outside the model, it seems that Duhem’s paradox is evident, and viewed here as a necessary (even important) component of interpretative practice.

On the question of whether regression results represent discoveries, some interviewees went around in circles rather than answering definitively. Interviewees with economic or epidemiological training were generally reluctant to rule out potential for discovery, as memories of previous analyses or the certainty of seminal findings drew interviewees toward the conclusion that coefficients represent real relationships. However, persistent, nagging awareness of bias and error prevented epidemiologists and economists from settling on this answer. Three illustrative extracts from academics trained in economics, medicine and epidemiology are provided in Table 3.

**Table 3:** Some interviewees articulated a position incorporating both discovery and construction

Do model estimates provide discoveries, or represent a construction?	Interviewee
I am more on the ‘trying to find what’s real’ side […][But] it’s hard [for the coefficient] to ever be truth when we have so many caveats with measurement, and whatever. I hope I’m at least getting closer to the truth, as I’m building. But then, […] I guess I am ‘building’ […] Those estimates inform interventions, so let’s hope there is some truth to it. […] I’m hedging, I’m in between. Because, of course, it’s not perfectly true.	Associate Professor, USA
We know from regression, if you’ve left something out of your model, you are not going to get perfect identification of what’s in the model. So, yeah, I suppose I don’t generally view… [pause] Or do I? […] I’ve definitely changed my view about the world [following a regression analysis], my priors have changed. So in that way I would be in the first camp [model is discovery], but, in general, I am in the second camp [model is construction].	Professor, UK
Smoking does cause lung cancer. You’d really have to be philosophically nimble to say that that is somehow not a generalisable fact. You would bloody well hope that your case control study, or cohort study picks it up, and your model is therefore measuring something that is true. But it will have error, due to statistical imprecision, as well as the three sources of error: confounding, selection bias and measurement error.	Professor, AU/NZ

For the first interviewee, technical caveats prevent coefficients representing straightforward truths. This interviewee finds themselves ‘in between’ awareness of the limitations of the model, and the sense that (in the long-run) regression methods generate reliable representations. For the second interviewee, technical limitations mean results aren’t perfect, but, sometimes, regression coefficients alter a researcher’s understanding of the world. (Identification with both ‘camps’ is perhaps Duhem’s paradox once again, as multiple interpretations of surprising results are available.)

The third extract is an example of seminal findings being held up to indicate that regression models detect facts. Duhem’s paradox is seemingly evaded via acknowledgement that regression models access true relationships, but reflect them imperfectly. References to smoking and lung cancer were widespread, but interviewees employing this example seemed to ignore heated debate surrounding the original claim, and the range of perspectives and methods contributing to final resolution. If results are known in advance it does seem straightforward that a model is ‘measuring something that is true’ as the interviewee in Table 3 indicates. Latour & Woolgar [22] noted in their study of molecular biologists that such distinction is possible only in retrospect, after a statement has stabilized as a fact. The splitting of ‘truth’ and ‘imperfect-statement-about-truth’ can occur only after controversy settles, after a statement like “smoking causes lung cancer” stabilizes within the agonistic field and is accepted by all concerned parties.

### Regression as knowledge construction

Several interviewees outlined a contrasting position, that regression models are not direct representations. Reasons why regression methods fail to capture or reflect ‘truth’ varied across two disciplinary groups. Social scientists tended to be reflexive and emphasize the role of their own values and choices

It is a representation that you know as a researcher you are constructing. Your own principles and kind of epistemological position, of course, are vital for how you’ve gone about that process. […] it is not like there is some sort of definitive truth out there, it is about thinking about defining a question and your own values, and the decision-making that you implement throughout the research process (Professor, UK).

As was just discussed, interviewees with epidemiology and economic backgrounds were more likely to locate lack of correspondence with reality within the *technical limits* of regression methods, rather than with the researcher:

It’s an imperfect representation of reality, because there is always confounding. Also, the variables and instruments themselves are imperfect, there are no perfect ways of measuring (Professor, UK).

Nine interviewees specifically discounted the likelihood of a single regression providing a durable answer.

That would be problematic, if people imagine they’re going to plunge into a dataset and ‘prove’ conclusively, forever, that this is the ‘right’ answer (Emeritus Professor, UK).

A similar idea was that coefficients help researchers arrive at conclusions. One statistician was adamant that coefficients do not answer research questions, they form *part* of the answer.

They represent help. I think they represent help to answer my questions. So, they represent possible effects of exposures on the outcomes. […]

Author: They help you to answer the question, they are not themselves the answer to the question?

The coefficients themselves? No. (Professor, UK).

This last view represents another route to the conclusion that it is researchers who answer questions and assign meaning, not models. If regression coefficients contribute to an answer (rather than *being* answers), this implies that regression models do not fully capture or accurately reflect reality. Ten interviewees expressed this view, suggesting variously that coefficients approximate true relationships:

I view it as **an approximation** to lots of these different relationships (Economics PhD).

Reflect some part or component of true relationships:

I think that it’s **one piece of reality** (Epidemiology PhD).

Approach true relationships from a certain direction (implying there are other directions):

They are a depiction of **some view of reality** (Epidemiology PhD).

Or are inherently limited:

They will **never fully describe reality** (Health Behaviour PhD).

Therefore, for interviewees who view regression as discovery, and those who view it as construction, regression coefficients are far from definitive or conclusive (just two interviewees reported that they fully base their interpretation on p-values, according to the NHST framework). For most interviewees, regression coefficients were not viewed as complete or authoritative statements about health. This contrasts with teaching materials wherein statistical results are very often presented as straightforward representations, subject to rule-based interpretations. In practice, it appears that regression coefficients are not understood as mechanically objective by this group of population health researchers.

### “It depends”

An obvious next question relates to how interviewees navigate this uncertainty to draw conclusions. Interviewees reported looking in a variety of places for confirmation that results have meaning (or do not have meaning), presented in Table 4.

**Table 4:** Places interviewees looked for confirmation that a coefficient has meaning

Study Feature	Illustrative Extract
How the model is setup	We make decisions in model building […] in what we ask, the tools we use, how we manage data, how we code data, there are choices that are subjective. Professor, USA
Study Design	It depends on the research design. If I participated in a randomized controlled trial I would feel differently about this than when I do a quasi-experiment, compared to doing a descriptive paper. Professor, Netherlands
Research Question	It partly depends on the model, and perhaps the questions you’re trying to ask with it. Professor, UK
Data Quality & Availability	The underlying data need to be good. Assistant Professor, Netherlands

When study design is perceived as appropriate, data as being of high-quality, and research questions appropriate for investigation via regression, interviewees described themselves as being more likely to accept a coefficient. However, any single item in Table 4 failing to meet expectations could lead to a result being dismissed. This seems to evoke the agonistic field as described by Latour & Woolgar [22]; new claims are reportedly treated with scepticism by default, and the study features presented in Table 4 represent examples of modalities; modifying or qualifying statements which tie results to the circumstances of their estimation, limiting their generalisability. This perhaps explains why so many interviewees reported assuming that regression coefficients do not perfectly or fully reflect reality. Status as reflecting truth may be reserved for the (rare) cases when the full range of factors in Table 4 are satisfied, for coefficients which are modality-free.

The features outlined in Table 4 were remarkably consistent across disciplines, and across continents. Economists, epidemiologists, health promotion researchers and social scientists highlighted these same features, suggesting that whilst diversity exists in researchers’ overall interpretative approach, there is broad consensus on the set of features which - generally - underpin the strength of any given regression analysis.

## Discussion

Interview data from 45 population health academics generally indicated that the meaning of a regression coefficient is not an inherent property of the coefficient, but lies in the eye of the beholder. Rather than being understood as mechanically objective, researchers reported considering a wide range of factors when interpreting regression output, including: knowledge from outside the model, whether results are expected or unexpected, common-sense, technical limitations, study design, the influence of the researcher, the research question, data quality and data availability. Regression coefficients appear subject to the uncertainty implied by Duhem’s paradox, and to agonistic processes prior to being accepted as valid.

In short, it is the researcher (and their readers) who imbue findings with meaning and differentiate ‘excalibur’ from error. This conclusion is consistent with established findings within the sociology of knowledge, history of science, and science studies. Scientific objects are not accompanied by a ‘halo’ conveying their meaning [40, 41]. Rather, interpretation is something scientists learn to do, in a particular scientific context, within a particular scientific culture [42]. Empirical confirmation that this view includes statistical objects is important, as in many contexts statistics have privileged epistemic status [43] and are assumed to be disconnected from the influence of social forces [44].

### Acknowledging uncertainty and the ‘seeing eye’

Dominant images of science are drawn from published manuscripts and textbooks. However these are simplified views, described by Thomas Kuhn as being “no more likely to fit the enterprise[…] than an image of national culture drawn from a tourist brochure” [30]. Whilst teaching materials and manuscripts present model development and interpretation of statistical coefficients via technical definitions (“average change in Y per unit-change in X”), interviewees in this study almost universally agreed that interpretation is more complex, and is a nuanced and iterative process requiring experience, and expertise.

This is not a new tension. In the late 19th Century, issues with the novel X-Ray technology were exposing clinicians to medico-legal risk. Clinicians reported that “we cannot always believe what we see, or rather fail to see” [45] and that the promising new equipment “may be easily made to tell untruth” [46] about health. Despite radiating authority, mechanical scientific representations often do require practitioners to wrestle with their potential deceptiveness [22, 23, 47]. In this study, interviewees were likewise aware that regression coefficients may reflect a distorted or incomplete view of reality, and, like 19th Century radiographers, reported drawing upon subject-matter knowledge and experience to draw conclusions, despite prevailing uncertainty.

### Practical implications: beyond simplistic views of biostatistical practice

The chief practical implications of this study are threefold. First, variation apparent among interviewees did not arise because some researchers *misunderstand* regression coefficients, but because there appear to be diverse epistemologies and approaches to knowledge construction at play within population health. This diversity complicates efforts to change and improve practice, because results suggest that both good and poor biostatistical choices are made in the context of a nuanced and (at least partly) subjective process which is (i) iterative and (ii) incorporates deeply-held beliefs about the purpose and limits of empirical enquiry. This perhaps explains why simplistic directives from statistical experts have failed to achieve widespread change. Efforts to improve practice, and calls to ‘change how we work’ [3] must be better attuned to this reality. The institutional, disciplinary and individual drivers of statistical choice-making are poorly understood, but must be reckoned with to avoid simplifying the task researchers attempt when they move from raw data to draft manuscript.

Second, it is significant that the majority of senior academics interviewed did not describe regression coefficients as being straightforward ‘answers’ to research questions, or as complete reflections of underlying relationships. This sceptical lens through which researchers view their own results is rarely discussed, or demonstrated in introductory statistics courses. Results from this study suggest that student-scientists require much more than familiarity with technical aspects of regression modelling in order to critically evaluate regression output. Additionally, results underscore the importance of standardised reporting guidelines such as STROBE [48] and CONSORT [49], as these frameworks provide reviewers and readers with detail regarding the full range of factors which inform interpretation.

Finally, if researchers apply different criteria to results which ‘make sense’ (or align with common-sense) compared with those which do not, this suggests that the agonistic processes surrounding regression results may vary in intensity depending upon researchers’ prior understandings and expectations. This finding requires further empirical exploration, but indicates that venues and mechanisms supporting quantitative researchers to reflect upon their own practice would be of substantial value, to encourage consideration and scrutiny of the ways these choices impact conclusions drawn from data.

Unfortunately, whilst results reinforce the central importance of technical caveats and qualifying statements in the minds of senior researchers, few scientific spaces actively support or encourage these kinds of discussions. The increasing pace of academia leaves many scholars within public health lacking space for reflection and deep work [50]. In publication, pressure to smooth over inconsistencies and produce ‘perfect’ results sections leaves some researchers reluctant to disclose such details [32, 51]. Some major journals (e.g. Nature [52]) have moved the method section to the end of peer-reviewed articles, perhaps reflecting the extent to which nuanced methodological detail is becoming devalued within some research contexts. In policy settings, technical caveats and qualifying statements like those attached to regression results by participants in this study have been reported as being routinely stripped away, positioned as inconvenient barriers to political action [53]. Collectively, these trends form the cultural backdrop to population health research. If transparent discussion of methodological issues counts against authors (in promotion, policy-uptake, or peer-review) then ‘tourist-brochure’ reporting of regressions will persist, even if teaching materials acquaint emerging scholars with the messy reality of analysis.

### Limitations

This study was limited to researchers sharing an interest in health equity. Randomised controlled trials are very challenging to conduct in this area [54], and so results are likely to apply primarily to observational study designs. Interviewees are predominantly late-career scholars, and representation from south America and Asia was lacking. These features of the study may limit generalisability of findings. Nevertheless, the inclusion of interviewees from diverse disciplines, countries and continents is a key strength, unusual in studies of academics [55]. Whilst interviewees identified a range of factors considered when interpreting coefficients, interview data did not provide a sense of their relative importance, or additive significance. Future studies could directly address these questions, including whether and how these factors are ranked in importance by researchers of varying geo-disciplinary backgrounds. Finally, this study investigates diversity in epistemic stance and does not necessarily reflect how researchers and their teams analyse data.

## Conclusion

Results from this study suggest that, in practice, the interpretation of regression coefficients is not as straightforward as textbooks and published articles indicate. Attempts to influence the conduct and presentation of regression models in published work should be attuned to the myriad factors which inform their interpretation.

## Data Availability

The qualitative data generated and analysed during the current study are not publicly available due to their identifying nature. Interviewees consented to publication of extracts from interviews, but not to release of the full transcripts. Further context around included data extracts is available from the corresponding author, on reasonable request.
